# 2-Amino-3-(hy­droxy­meth­yl)pyridinium 2-benzoyl­benzoate monohydrate

**DOI:** 10.1107/S1600536812005612

**Published:** 2012-02-17

**Authors:** Hakkı Yasin Odabaşoğlu, Orhan Büyükgüngör, Osman Ozan Avinç, Mustafa Odabaşoğlu

**Affiliations:** a Department of Textile Engineering, Faculty of Engineering, Pamukkale University, TR-20070 Kınıklı Denizli, Turkey; b Department of Physics, Faculty of Arts and Science, Ondokuz Mayıs University, TR-55139 Kurupelit Samsun, Turkey; c Chemistry Programme, Denizli Higher Vocational School, Pamukkale University, TR-20159 Denizli, Turkey

## Abstract

In the title hydrated salt, C_6_H_9_N_2_O^+^·C_14_H_9_O_3_^−^·H_2_O, the dihedral angle between the benzene rings of the 2-benzoyl­benzoate anion is 82.04 (14)°, while the angles between the aromatic ring of the pyridinium cation and each of the benzene rings of the anion are 4.42 (14) and 82.04 (14)°. In the crystal, mol­ecules are linked by N—H⋯O and O—H⋯O hydrogen bonds, generating a three-dimensional network with *R*_2_^2^(8), *R*_6_^6^(16) and *R*_4_^4^(6) motifs. The crystal packing is further stabilized by two π–π inter­actions, one between pyridinium rings, and another between the benzene benzoate and pyridinium rings of neighbouring mol­ecules, with centroid-to-centroid distances of 3.559 (2) and 3.606 (2) Å, respectively.

## Related literature

For general background, see: Lehn (1990 ▶); Mrozek & Glowiak (2004 ▶); Yang *et al.* (1995 ▶); Goswami & Ghosh (1997 ▶); Goswami *et al.* (1998 ▶); Lah *et al.* (2001 ▶); Hong & Sun (2008 ▶). For related structures, see: Büyükgüngör & Odabaşoğlu (2002 ▶); Büyükgüngör *et al.* (2004 ▶); Odabaşoğlu & Büyükgüngör (2007 ▶, 2008 ▶); Odabaşoğlu *et al.* (2003*b* ▶,*c* ▶). For the synthesis of the title compound, see: Odabaşoğlu *et al.* (2003*a* ▶). For ring-motif details, see: Bernstein *et al.* (1995 ▶); Etter (1990 ▶).
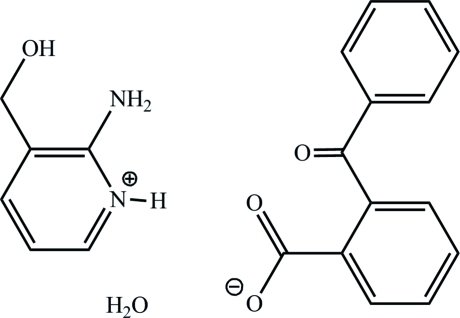


## Experimental

### 

#### Crystal data

C_6_H_9_N_2_O^+^·C_14_H_9_O_3_^−^·H_2_O
*M* *_r_* = 368.38
Monoclinic, 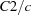

*a* = 15.9259 (11) Å
*b* = 8.4898 (4) Å
*c* = 27.6362 (19) Å
β = 93.468 (5)°
*V* = 3729.8 (4) Å^3^
*Z* = 8
Mo *K*α radiation
μ = 0.10 mm^−1^
*T* = 296 K
0.35 × 0.30 × 0.26 mm

#### Data collection

Stoe IPDS 2 diffractometer
Absorption correction: integration (*X-RED32*; Stoe & Cie, 2002 ▶) *T*_min_ = 0.967, *T*_max_ = 0.976
9399 measured reflections
3523 independent reflections
1792 reflections with *I* > 2σ(*I*)
*R*_int_ = 0.117

#### Refinement

*R*[*F* ^2^ > 2σ(*F* ^2^)] = 0.062
*wR*(*F* ^2^) = 0.156
*S* = 0.95
3503 reflections
254 parameters
4 restraints
H atoms treated by a mixture of independent and constrained refinement
Δρ_max_ = 0.18 e Å^−3^
Δρ_min_ = −0.21 e Å^−3^

### 

Data collection: *X-AREA* (Stoe & Cie, 2002 ▶); cell refinement: *X-AREA*; data reduction: *X-RED32* (Stoe & Cie, 2002 ▶); program(s) used to solve structure: *SHELXS97* (Sheldrick, 2008 ▶); program(s) used to refine structure: *SHELXL97* (Sheldrick, 2008 ▶); molecular graphics: *ORTEP-3 for Windows* (Farrugia, 1997 ▶); software used to prepare material for publication: *WinGX* (Farrugia, 1999 ▶).

## Supplementary Material

Crystal structure: contains datablock(s) I, global. DOI: 10.1107/S1600536812005612/lr2047sup1.cif

Structure factors: contains datablock(s) I. DOI: 10.1107/S1600536812005612/lr2047Isup2.hkl

Supplementary material file. DOI: 10.1107/S1600536812005612/lr2047Isup3.cml

Additional supplementary materials:  crystallographic information; 3D view; checkCIF report

## Figures and Tables

**Table 1:** Hydrogen-bond geometry (Å, °)

*D*—H⋯*A*	*D*—H	H⋯*A*	*D*⋯*A*	*D*—H⋯*A*
N1—H1⋯O1^i^	0.86	1.93	2.775 (3)	167
N1—H1⋯O2^i^	0.86	2.62	3.303 (3)	137
N2—H2*A*⋯O2^i^	0.86	2.04	2.845 (3)	156
N2—H2*B*⋯O5^ii^	0.86	2.11	2.942 (4)	162
O4—H4*A*⋯O2^iii^	0.86 (2)	1.92 (2)	2.765 (3)	168 (4)
O5—H5*A*⋯O4	0.86 (2)	1.96 (2)	2.807 (3)	167 (4)
O5—H5*B*⋯O1	0.86 (2)	2.28 (4)	2.964 (4)	136 (4)

Symmetry codes: (i) 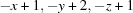; (ii) 
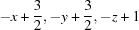
; (iii) 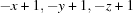.

**Fig. 1.**
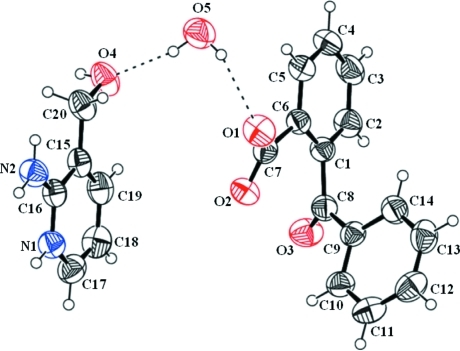
A view of (I). Displacement ellipsoids are drawn at the 40% probability level.

**Fig. 2.**
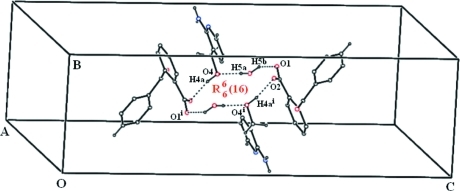
A partial view of the packing of (I), showing the formation of O—H···O bonded R66(16) motif [Symmetry code:(i) 1 - x, 1 - y, 1 - z]

**Fig. 3.**
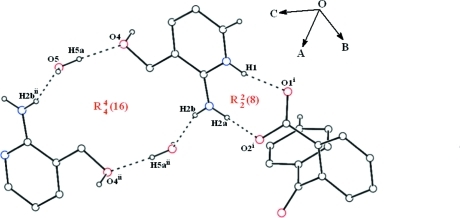
A partial view of the packing of (I) with hydrogen bonded R22(8) and R44(16) motifs [Symmetry codes:(i) 3/2 - x, 3/2 - y, 1 - z; (ii) x + 1/2, y - 1/2, z].

**Fig. 4.**
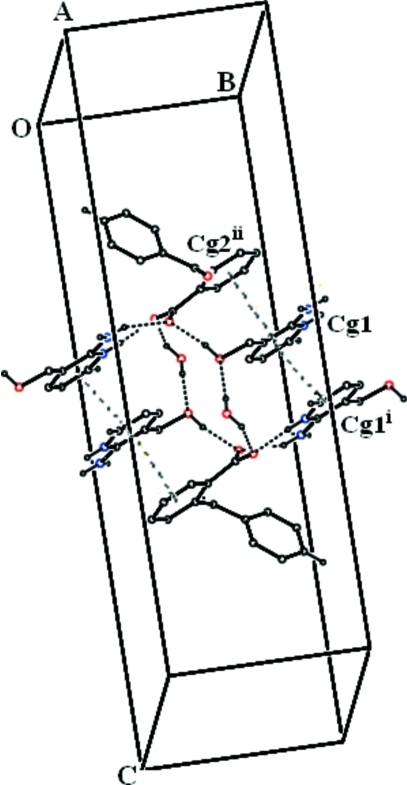
A partial view of the packing of (I), showing the formation of π–π interactions. [Symmetry codes:(i) 1 - x, 3/2 - y, 1 - z; (ii) 1 - x, 1 - y, 1 - z].

## supplementary crystallographic information

### Comment

The cotton fabrics which have been treated with benzophenone derivatives have powerful antibacterial properties against *S. aureus* and *E. coli*, and benzoylbenzoic acid derivatives treated cotton fabric demonstrated pesticide degradation ability, under UV irradiation (Hong & Sun, 2008). Furthermore, the copper(II) complexes of 2-aminopyridinium carboxylates have important properties in the applications of pharmaceuticals, fungicides, oxygen transfer, oxidative addition, homogenous hydrogenation, gas occlusion compounds, and solvent extractions processes (Yang *et al.*, 1995; Lah *et al.*, 2001). Hydrogen bonding plays a key role in molecular recognition (Goswami & Ghosh, 1997) and crystal engineering research (Goswami *et al.*, 1998). The design of highly specific solid-state structures is of considerable significance in organic chemistry due to their important applications in the development of new optical, magnetic and electronic systems (Lehn, 1990). With this in mind, we had aimed the synthesis of (*E*)-2-(3-(hydroxymethyl)pyridin-2-ylimino)(phenyl) methyl)benzoic acid by reaction of 2-Amino-3-hydroxymethylpyridine and 2-benzoylbenzoic acid. But this compound can not be produced in the reaction conditions (Odabaşoǧlu *et al.*, 2003*a*), instead the title compound was obtained (Scheme 1, Fig. 1).

The crystal structure of the title compound exhibit four N—H···O, three O—H···O hydrogen bonds and two π–π interactions. The C—H···O hydrogen bonds generate *R*_6_^6^(16) hydrogen bonded motifs (Fig. 2) (Bernstein *et al.*, 1995; Etter, 1990). Pyridinium ions, H_2_O molecules and carboxylate ions give edge fused *R*_4_^4^(16) *R*_2_^2^(8) hydrogen bonded rings by O—H···O and N—H···O hydrogen bonds (Fig. 3).

The dihedral angle between the benzene rings B:(C1–C6) and C:(C9–C14) of the 2-benzoylbenzoate fragment is 82.04 (14)°, while the angles between the aromatic ring A:(C15,C16,N1,C17,C18,C19) of the 2-amino-3-hydroxymethylpiridinium fragment with each of them are 4.42 (14)° and 82.04 (14)° respectively. There are also stacking interactions between the A–A and A–B rings of symmetry-related molecules, with centroid–centroid distance of 3.559 (2) Å and 3.606 (2) Å, respectively (Fig. 4). The bond distance and angles in (I) are expected value and consistent with the literature (Mrozek & Glowiak, 2004; Büyükgüngör & Odabaşoǧlu, 2002; Odabaşoğlu *et al.*, 2003*b*,*c*; Büyükgüngör *et al.*, 2004; Odabaşoğlu & Büyükgüngör, 2007, 2008).

### Experimental

The title compound is obtained by reaction of 2-Amino-3-hydroxymethylpyridine and 2-benzoylbenzoic acid under the experimental condition previously described (Odabaşoǧlu *et al.* 2003*a*). Suitable crystal of the title compound were obtained by slow evaporation from a solution of the reaction mixture in ethanol.

### Refinement

H atoms bonded to O were located in a difference map and refined isotropically. Constrained C—H and N—H bond lengths and isotropic U parameters: 0.93 Å and *U*_iso_(H) = 1.2*U*_eq_(C) for aromatic C—H; 0.97 Å and *U*_iso_(H) = 1.2*U*_eq_(C) for methylene C—H; 0.86 Å and *U*_iso_(H) = 1.2*U*_eq_(N) for N—H.

### Crystal data

**Table:**

C_6_H_9_N_2_O^+^·C_14_H_9_O_3_^−^·H_2_O	*F*(000) = 1552
*M**_r_* = 368.38	*D*_x_ = 1.312 Mg m^−^^3^
Monoclinic, *C*2/*c*	Mo *K*α radiation, λ = 0.71073 Å
Hall symbol: -C 2yc	Cell parameters from 8608 reflections
*a* = 15.9259 (11) Å	θ = 1.5–26.1°
*b* = 8.4898 (4) Å	µ = 0.10 mm^−^^1^
*c* = 27.6362 (19) Å	*T* = 296 K
β = 93.468 (5)°	Block, colourless
*V* = 3729.8 (4) Å^3^	0.35 × 0.30 × 0.26 mm
*Z* = 8	

### Data collection

**Table:**

Stoe IPDS 2 diffractometer	3523 independent reflections
Radiation source: fine-focus sealed tube	1792 reflections with *I* > 2σ(*I*)
Graphite monochromator	*R*_int_ = 0.117
Detector resolution: 6.67 pixels mm^-1^	θ_max_ = 25.7°, θ_min_ = 1.5°
ω–scan rotation method	*h* = −19→15
Absorption correction: integration (*X-RED*; Stoe & Cie, 2002)	*k* = −9→10
*T*_min_ = 0.967, *T*_max_ = 0.976	*l* = −33→33
9399 measured reflections	

### Refinement

**Table:**

Refinement on *F*^2^	Secondary atom site location: difference Fourier map
Least-squares matrix: full	Hydrogen site location: inferred from neighbouring sites
*R*[*F*^2^ > 2σ(*F*^2^)] = 0.062	H atoms treated by a mixture of independent and constrained refinement
*wR*(*F*^2^) = 0.156	*w* = 1/[σ^2^(*F*_o_^2^) + (0.0625*P*)^2^] where *P* = (*F*_o_^2^ + 2*F*_c_^2^)/3
*S* = 0.95	(Δ/σ)_max_ < 0.001
3503 reflections	Δρ_max_ = 0.18 e Å^−^^3^
254 parameters	Δρ_min_ = −0.21 e Å^−^^3^
4 restraints	Extinction correction: *SHELXL97* (Sheldrick, 2008), Fc^*^=kFc[1+0.001xFc^2^λ^3^/sin(2θ)]^-1/4^
Primary atom site location: structure-invariant direct methods	Extinction coefficient: 0.0034 (5)

### Special details

**Table:**

Geometry. All e.s.d.'s (except the e.s.d. in the dihedral angle between two l.s. planes) are estimated using the full covariance matrix. The cell e.s.d.'s are taken into account individually in the estimation of e.s.d.'s in distances, angles and torsion angles; correlations between e.s.d.'s in cell parameters are only used when they are defined by crystal symmetry. An approximate (isotropic) treatment of cell e.s.d.'s is used for estimating e.s.d.'s involving l.s. planes.
Refinement. Refinement of *F*^2^ against ALL reflections. The weighted *R*-factor *wR* and goodness of fit *S* are based on *F*^2^, conventional *R*-factors *R* are based on *F*, with *F* set to zero for negative *F*^2^. The threshold expression of *F*^2^ > σ(*F*^2^) is used only for calculating *R*-factors(gt) *etc*. and is not relevant to the choice of reflections for refinement. *R*-factors based on *F*^2^ are statistically about twice as large as those based on *F*, and *R*-factors based on ALL data will be even larger.

### Fractional atomic coordinates and isotropic or equivalent isotropic displacement parameters (Å^2^)

**Table:**

	*x*	*y*	*z*	*U*_iso_*/*U*_eq_	
C1	0.40651 (19)	0.3876 (3)	0.66050 (9)	0.0562 (7)	
C2	0.4022 (2)	0.2387 (4)	0.68081 (10)	0.0692 (9)	
H2	0.3510	0.2001	0.6902	0.083*	
C3	0.4738 (3)	0.1478 (4)	0.68716 (11)	0.0788 (10)	
H3	0.4705	0.0477	0.7006	0.095*	
C4	0.5489 (2)	0.2034 (4)	0.67396 (11)	0.0755 (10)	
H4	0.5970	0.1420	0.6788	0.091*	
C5	0.5540 (2)	0.3510 (4)	0.65331 (10)	0.0653 (8)	
H5	0.6058	0.3881	0.6443	0.078*	
C6	0.48330 (18)	0.4444 (3)	0.64586 (8)	0.0542 (7)	
C7	0.4857 (2)	0.5976 (4)	0.61819 (9)	0.0591 (8)	
C8	0.32807 (19)	0.4875 (4)	0.65745 (9)	0.0595 (8)	
C9	0.32802 (18)	0.6335 (4)	0.68740 (9)	0.0553 (7)	
C10	0.27551 (19)	0.7577 (4)	0.67355 (11)	0.0652 (8)	
H10	0.2441	0.7532	0.6441	0.078*	
C11	0.2694 (2)	0.8874 (4)	0.70280 (13)	0.0767 (10)	
H11	0.2342	0.9705	0.6931	0.092*	
C12	0.3152 (2)	0.8940 (5)	0.74628 (13)	0.0819 (10)	
H12	0.3106	0.9812	0.7663	0.098*	
C13	0.3682 (2)	0.7722 (5)	0.76063 (11)	0.0818 (10)	
H13	0.3990	0.7771	0.7903	0.098*	
C14	0.3757 (2)	0.6423 (4)	0.73080 (10)	0.0689 (9)	
H14	0.4126	0.5613	0.7400	0.083*	
O1	0.55266 (16)	0.6753 (3)	0.61989 (8)	0.0810 (7)	
O2	0.41975 (14)	0.6353 (2)	0.59401 (7)	0.0673 (6)	
O3	0.26539 (15)	0.4449 (3)	0.63399 (8)	0.0821 (7)	
C15	0.5523 (2)	0.8106 (3)	0.45397 (9)	0.0591 (8)	
C16	0.5503 (2)	0.9647 (4)	0.43366 (9)	0.0580 (7)	
C17	0.4025 (2)	0.9701 (5)	0.43705 (11)	0.0734 (9)	
H17	0.3523	1.0245	0.4307	0.088*	
C18	0.4010 (2)	0.8251 (5)	0.45727 (12)	0.0778 (10)	
H18	0.3508	0.7790	0.4653	0.093*	
C19	0.4776 (2)	0.7474 (4)	0.46559 (10)	0.0730 (9)	
H19	0.4776	0.6480	0.4797	0.088*	
C20	0.6347 (2)	0.7288 (4)	0.46227 (11)	0.0695 (9)	
H20A	0.6627	0.7266	0.4321	0.083*	
H20B	0.6698	0.7890	0.4855	0.083*	
N1	0.47595 (17)	1.0371 (3)	0.42587 (8)	0.0639 (7)	
H1	0.4748	1.1297	0.4133	0.077*	
N2	0.61927 (17)	1.0410 (3)	0.42175 (8)	0.0683 (7)	
H2A	0.6152	1.1340	0.4094	0.082*	
H2B	0.6678	0.9973	0.4264	0.082*	
O4	0.62787 (17)	0.5725 (3)	0.47963 (7)	0.0814 (7)	
H4A	0.619 (3)	0.514 (4)	0.4545 (11)	0.122*	
O5	0.70194 (16)	0.5481 (4)	0.57409 (9)	0.0921 (8)	
H5A	0.673 (3)	0.560 (5)	0.5473 (9)	0.138*	
H5B	0.682 (3)	0.614 (5)	0.5941 (12)	0.138*	

### Atomic displacement parameters (Å^2^)

**Table:**

	*U*^11^	*U*^22^	*U*^33^	*U*^12^	*U*^13^	*U*^23^
C1	0.0573 (19)	0.0569 (18)	0.0540 (14)	0.0035 (15)	−0.0003 (12)	−0.0019 (13)
C2	0.071 (2)	0.064 (2)	0.0729 (18)	−0.0044 (19)	0.0066 (16)	0.0111 (15)
C3	0.099 (3)	0.065 (2)	0.0708 (19)	0.008 (2)	−0.0016 (18)	0.0184 (16)
C4	0.077 (3)	0.077 (2)	0.0709 (18)	0.019 (2)	−0.0068 (17)	0.0096 (17)
C5	0.060 (2)	0.073 (2)	0.0624 (16)	0.0054 (18)	−0.0011 (14)	0.0028 (15)
C6	0.0581 (19)	0.0530 (17)	0.0508 (13)	0.0011 (16)	−0.0013 (12)	0.0000 (12)
C7	0.060 (2)	0.065 (2)	0.0524 (14)	−0.0022 (18)	0.0044 (14)	−0.0041 (13)
C8	0.0519 (19)	0.069 (2)	0.0577 (15)	−0.0028 (16)	0.0015 (14)	0.0013 (14)
C9	0.0474 (17)	0.0605 (18)	0.0587 (15)	−0.0024 (15)	0.0077 (12)	−0.0002 (13)
C10	0.0555 (19)	0.070 (2)	0.0700 (17)	0.0042 (18)	0.0025 (14)	−0.0030 (16)
C11	0.064 (2)	0.065 (2)	0.102 (3)	0.0034 (18)	0.0106 (18)	−0.0143 (18)
C12	0.077 (3)	0.080 (2)	0.090 (2)	−0.004 (2)	0.0145 (19)	−0.0252 (19)
C13	0.086 (3)	0.098 (3)	0.0616 (17)	−0.011 (2)	0.0015 (17)	−0.0127 (19)
C14	0.072 (2)	0.072 (2)	0.0623 (17)	0.0015 (18)	−0.0006 (15)	0.0027 (16)
O1	0.0715 (16)	0.0775 (16)	0.0936 (15)	−0.0159 (14)	0.0010 (12)	0.0102 (12)
O2	0.0696 (15)	0.0594 (13)	0.0715 (11)	0.0024 (11)	−0.0063 (11)	0.0074 (10)
O3	0.0630 (15)	0.0853 (16)	0.0960 (15)	0.0009 (14)	−0.0107 (12)	−0.0177 (13)
C15	0.074 (2)	0.0551 (17)	0.0477 (13)	−0.0081 (17)	−0.0011 (13)	0.0004 (13)
C16	0.065 (2)	0.0583 (19)	0.0498 (14)	−0.0058 (18)	0.0005 (13)	−0.0007 (13)
C17	0.064 (2)	0.087 (3)	0.0703 (18)	−0.003 (2)	0.0061 (15)	−0.0099 (18)
C18	0.073 (3)	0.082 (3)	0.0789 (19)	−0.019 (2)	0.0095 (17)	−0.0024 (19)
C19	0.088 (3)	0.069 (2)	0.0620 (17)	−0.013 (2)	0.0050 (16)	0.0052 (15)
C20	0.088 (2)	0.0548 (19)	0.0637 (16)	−0.0058 (18)	−0.0081 (16)	0.0051 (14)
N1	0.0685 (18)	0.0597 (16)	0.0633 (13)	−0.0001 (15)	0.0019 (12)	0.0000 (12)
N2	0.0722 (18)	0.0584 (16)	0.0741 (15)	−0.0010 (15)	0.0036 (13)	0.0116 (13)
O4	0.118 (2)	0.0554 (13)	0.0679 (12)	−0.0034 (14)	−0.0144 (13)	0.0085 (10)
O5	0.0718 (17)	0.113 (2)	0.0909 (16)	0.0031 (16)	0.0009 (13)	0.0184 (15)

### Geometric parameters (Å, º)

**Table:**

C1—C6	1.397 (4)	C13—C14	1.386 (5)
C1—C2	1.386 (4)	C13—H13	0.9300
C1—C8	1.508 (4)	C14—H14	0.9300
C2—C3	1.379 (5)	C15—C19	1.361 (4)
C2—H2	0.9300	C15—C16	1.423 (4)
C3—C4	1.356 (5)	C15—C20	1.491 (5)
C3—H3	0.9300	C16—N2	1.333 (4)
C4—C5	1.382 (5)	C16—N1	1.340 (4)
C4—H4	0.9300	C17—N1	1.353 (4)
C5—C6	1.383 (4)	C17—C18	1.353 (5)
C5—H5	0.9300	C17—H17	0.9300
C6—C7	1.511 (4)	C18—C19	1.393 (5)
C7—O1	1.252 (4)	C18—H18	0.9300
C7—O2	1.252 (3)	C19—H19	0.9300
C8—O3	1.212 (3)	C20—O4	1.417 (4)
C8—C9	1.490 (4)	C20—H20A	0.9700
C9—C14	1.382 (4)	C20—H20B	0.9700
C9—C10	1.385 (4)	N1—H1	0.8600
C10—C11	1.373 (4)	N2—H2A	0.8600
C10—H10	0.9300	N2—H2B	0.8600
C11—C12	1.369 (5)	O4—H4A	0.859 (19)
C11—H11	0.9300	O5—H5A	0.858 (18)
C12—C13	1.378 (5)	O5—H5B	0.858 (19)
C12—H12	0.9300		
			
C6—C1—C2	119.8 (3)	C12—C13—C14	119.9 (3)
C6—C1—C8	121.8 (3)	C12—C13—H13	120.0
C2—C1—C8	118.3 (3)	C14—C13—H13	120.0
C3—C2—C1	120.1 (3)	C9—C14—C13	119.8 (3)
C3—C2—H2	120.0	C9—C14—H14	120.1
C1—C2—H2	120.0	C13—C14—H14	120.1
C4—C3—C2	120.4 (3)	C19—C15—C16	117.2 (3)
C4—C3—H3	119.8	C19—C15—C20	123.7 (3)
C2—C3—H3	119.8	C16—C15—C20	119.1 (3)
C3—C4—C5	120.1 (3)	N2—C16—N1	118.1 (3)
C3—C4—H4	119.9	N2—C16—C15	123.1 (3)
C5—C4—H4	119.9	N1—C16—C15	118.8 (3)
C4—C5—C6	120.9 (3)	N1—C17—C18	120.9 (3)
C4—C5—H5	119.6	N1—C17—H17	119.5
C6—C5—H5	119.6	C18—C17—H17	119.5
C1—C6—C5	118.6 (3)	C17—C18—C19	117.5 (4)
C1—C6—C7	119.6 (3)	C17—C18—H18	121.2
C5—C6—C7	121.6 (3)	C19—C18—H18	121.2
O1—C7—O2	124.8 (3)	C15—C19—C18	122.9 (3)
O1—C7—C6	118.8 (3)	C15—C19—H19	118.5
O2—C7—C6	116.3 (3)	C18—C19—H19	118.5
O3—C8—C9	121.2 (3)	O4—C20—C15	113.8 (3)
O3—C8—C1	121.0 (3)	O4—C20—H20A	108.8
C9—C8—C1	117.7 (2)	C15—C20—H20A	108.8
C14—C9—C10	119.3 (3)	O4—C20—H20B	108.8
C14—C9—C8	120.5 (3)	C15—C20—H20B	108.8
C10—C9—C8	120.0 (3)	H20A—C20—H20B	107.7
C11—C10—C9	120.7 (3)	C16—N1—C17	122.6 (3)
C11—C10—H10	119.6	C16—N1—H1	118.7
C9—C10—H10	119.6	C17—N1—H1	118.7
C12—C11—C10	119.7 (3)	C16—N2—H2A	120.0
C12—C11—H11	120.1	C16—N2—H2B	120.0
C10—C11—H11	120.1	H2A—N2—H2B	120.0
C11—C12—C13	120.5 (3)	C20—O4—H4A	106 (3)
C11—C12—H12	119.8	H5A—O5—H5B	106 (3)
C13—C12—H12	119.8		
			
C6—C1—C2—C3	0.9 (4)	C1—C8—C9—C10	155.1 (3)
C8—C1—C2—C3	−175.8 (3)	C14—C9—C10—C11	−1.1 (5)
C1—C2—C3—C4	0.5 (5)	C8—C9—C10—C11	174.4 (3)
C2—C3—C4—C5	−1.0 (5)	C9—C10—C11—C12	−0.4 (5)
C3—C4—C5—C6	0.1 (5)	C10—C11—C12—C13	0.8 (5)
C2—C1—C6—C5	−1.7 (4)	C11—C12—C13—C14	0.3 (5)
C8—C1—C6—C5	174.8 (2)	C10—C9—C14—C13	2.2 (5)
C2—C1—C6—C7	172.4 (3)	C8—C9—C14—C13	−173.3 (3)
C8—C1—C6—C7	−11.0 (4)	C12—C13—C14—C9	−1.9 (5)
C4—C5—C6—C1	1.2 (4)	C19—C15—C16—N2	178.5 (3)
C4—C5—C6—C7	−172.8 (3)	C20—C15—C16—N2	−0.4 (4)
C1—C6—C7—O1	155.1 (3)	C19—C15—C16—N1	−1.8 (4)
C5—C6—C7—O1	−30.9 (4)	C20—C15—C16—N1	179.4 (2)
C1—C6—C7—O2	−26.5 (4)	N1—C17—C18—C19	−0.7 (5)
C5—C6—C7—O2	147.4 (3)	C16—C15—C19—C18	1.7 (4)
C6—C1—C8—O3	121.8 (3)	C20—C15—C19—C18	−179.6 (3)
C2—C1—C8—O3	−61.6 (4)	C17—C18—C19—C15	−0.5 (5)
C6—C1—C8—C9	−63.1 (3)	C19—C15—C20—O4	5.2 (4)
C2—C1—C8—C9	113.5 (3)	C16—C15—C20—O4	−176.1 (2)
O3—C8—C9—C14	145.7 (3)	N2—C16—N1—C17	−179.5 (2)
C1—C8—C9—C14	−29.5 (4)	C15—C16—N1—C17	0.8 (4)
O3—C8—C9—C10	−29.8 (4)	C18—C17—N1—C16	0.5 (4)

### Hydrogen-bond geometry (Å, º)

**Table:**

*D*—H···*A*	*D*—H	H···*A*	*D*···*A*	*D*—H···*A*
N1—H1···O1^i^	0.86	1.93	2.775 (3)	167
N1—H1···O2^i^	0.86	2.62	3.303 (3)	137
N2—H2*A*···O2^i^	0.86	2.04	2.845 (3)	156
N2—H2*B*···O5^ii^	0.86	2.11	2.942 (4)	162
O4—H4*A*···O2^iii^	0.86 (2)	1.92 (2)	2.765 (3)	168 (4)
O5—H5*A*···O4	0.86 (2)	1.96 (2)	2.807 (3)	167 (4)
O5—H5*B*···O1	0.86 (2)	2.28 (4)	2.964 (4)	136 (4)

Symmetry codes: (i) −*x*+1, −*y*+2, −*z*+1; (ii) −*x*+3/2, −*y*+3/2, −*z*+1; (iii) −*x*+1, −*y*+1, −*z*+1.
